# Module of Axis-based Nexus Attention for weakly supervised object localization

**DOI:** 10.1038/s41598-023-45796-8

**Published:** 2023-10-30

**Authors:** Junghyo Sohn, Eunjin Jeon, Wonsik Jung, Eunsong Kang, Heung-Il Suk

**Affiliations:** 1 Department of Artificial Intelligence, Korea University, Seoul, 02841 Republic of Korea; 2 Department of Brain and Cognitive Engineering, Korea University, Seoul, 02841 Republic of Korea

**Keywords:** Computational science, Computer science

## Abstract

Weakly supervised object localization tasks remain challenging to identify and segment an entire object rather than only discriminative parts of the object. To tackle this problem, corruption-based approaches have been devised, which involve the training of non-discriminative regions by corrupting (e.g., erasing) the input images or intermediate feature maps. However, this approach requires an additional hyperparameter, the corrupting threshold, to determine the degree of corruption and can unfavorably disrupt training. It also tends to localize object regions coarsely. In this paper, we propose a novel approach, Module of Axis-based Nexus Attention (MoANA), which helps to adaptively activate less discriminative regions along with the class-discriminative regions without an additional hyperparameter, and elaborately localizes an entire object. Specifically, MoANA consists of three mechanisms (1) triple-view attentions representation, (2) attentions expansion, and (3) features calibration mechanism. Unlike other attention-based methods that train a coarse attention map with the same values across elements in feature maps, MoANA trains fine-grained values in an attention map by assigning different attention values to each element. We validated MoANA by comparing it with various methods. We also analyzed the effect of each component in MoANA and visualized attention maps to provide insights into the calibration.

## Introduction

During the last decade, various deep learning models have been developed for inferring the bounding box of objects in natural images, and have achieved remarkable performance in object localization^1–3^. However, from the perspective of data efficiency, those works used a fully-labeled dataset with respect to localization, which is regarded as a major limitation. The construction of such a dataset is time-consuming and labor-intensive leading to their limited applicability in practice.

Meanwhile, Weakly Supervised Object Localization (WSOL) methods employ only class labels, without using the target bounding box labels^4–19^. WSOL has therefore attracted considerable attention, because of its potential for training in a data-efficient manner. The main idea of WSOL is to detect the class-discriminative regions via an object recognition task, and to utilize those regions for the localization of the identified object.

A Class Activation Map (CAM)^4^, one of the representative methods in WSOL, estimates the class-specific discriminative regions based on the inferred class scores. However, various studies^5–19^ have addressed that CAM-based methods are not capable of capturing overall object regions in a finer way, because they focus only on the class-discriminative regions, disregarding non-discriminative regions. For this reason, many of the output bounding boxes are either over-sized or under-sized with respect to the target object. There have been efforts to tackle these challenges via diverse network architectures and learning strategies^5–21^.

Among the diverse WSOL strategies, a corruption approach is most commonly used. Corruption methods intentionally corrupt (e.g., erase) parts of an input image^6,11,19^ or feature map^9,13,17^. For the corruption methods, two different strategies are exploited: random corruption and network-guided corruption. The random corruption approach removes a small patch within an image at random and uses the corrupted image to learn richer feature representations^6,11,19^. This approach helps the trained network to discover diverse discriminative representations, thus detecting more object-related regions. The network-guided corruption approach adaptively corrupts feature maps by dropping out the most discriminative regions based on the integrated activation maps^9,13,17^. The corrupted feature maps only include non-discriminative regions, which enables localization by modifying the original feature map^13,17^, or making an activation map through an additional layer or network^9^.

**Figure 1:**
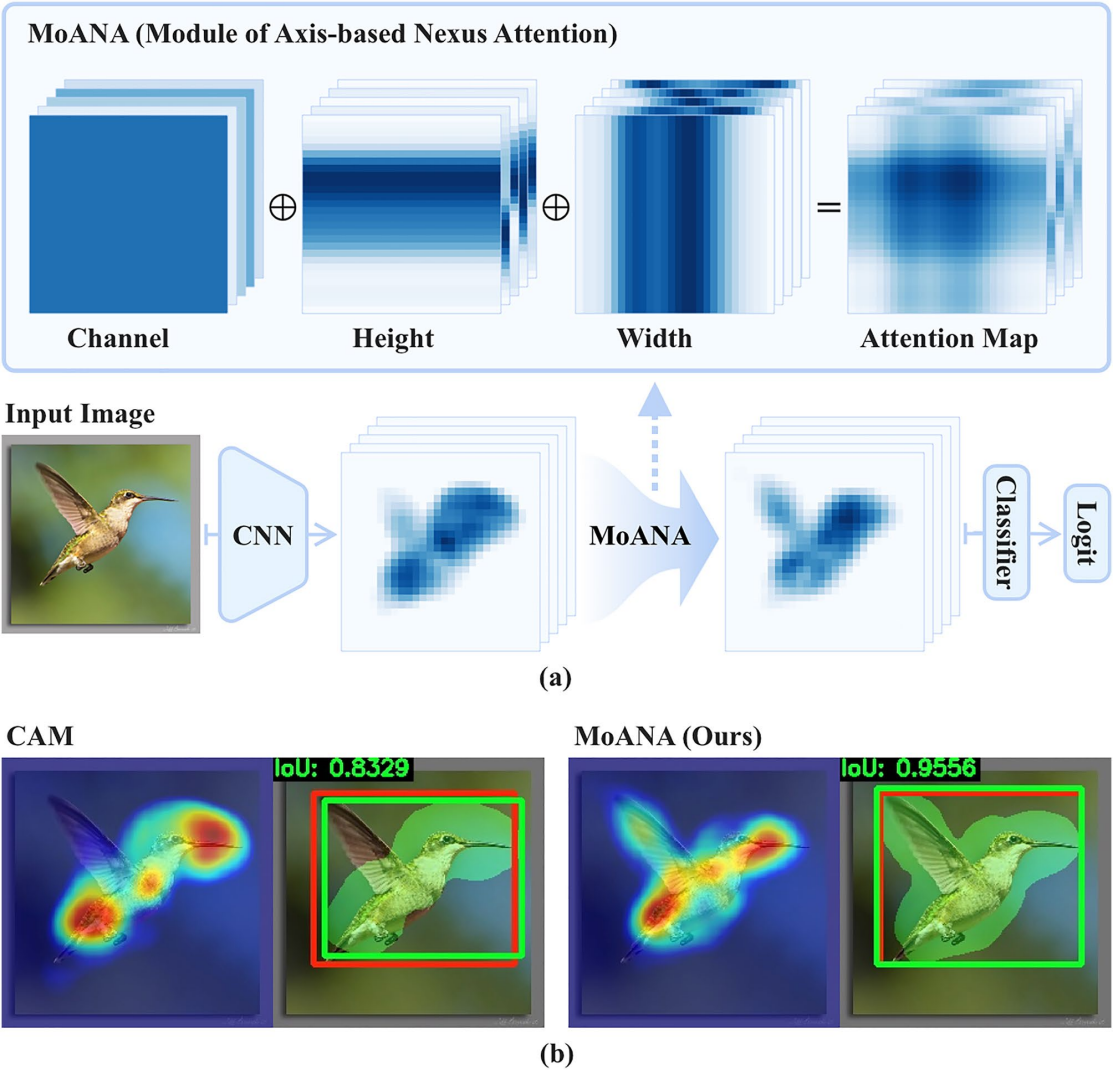
(**a**) Overview of our MoANA method, which generates fine-grained attended maps for WSOL by incorporating triple-view attentions (channel, height, and width) before a classifier. The full attention map is generated by an outer sum of the triple-view attentions. (**b**) Comparison of CAM^4^ and our MoANA with respect to an activation map (left) and a localization (right). In the localization, red and green boxes denote the ground-truth and predicted bounding boxes, respectively. The green-masked region indicates the activation map after applying a threshold. These maps were generated using Python 3.6.0, available at https://www.python.org.

While those methods improve the performance, they have limitations that should be further considered. First, the random-corruption approach^6,11^ potentially disrupts network learning due to unexpected information loss^9,13^. For example, if object-characteristic parts are removed from an input image, a network is enforced to discover other parts from the remaining regions. When there exists no discriminative region anymore, the network would be trained incorrectly. Second, the network-guided corruption approach^9,13,17^ introduces an additional hyperparameter (e.g., corrupting threshold) to determine the most discriminative regions. Also, most network-guided corruption methods use a specially designed module to generate an attention map in which the most discriminatory regions are hidden, to capture the integral extent of an object. However, it mainly exploits the coarse information in the channel or spatial attention and applies the same attention values to units in feature maps.

In this paper, we propose a novel Module of Axis-based Nexus Attention (MoANA), which accurately localizes object-related regions in an image. Specifically, we propose a new mechanism to generate a fine-level axis-based attention map that utilizes a series of information distributed over channels, heights, and widths with an attention mechanism towards calibrating features. The fine-level axis-based attention map is the same size as the input feature maps; thus, the attention is assigned for each unit across feature maps and channels. Compared to other existing methods, there is no need to mask patches in an image in our method. Further, we do not require an additional hyperparameter, such as a corrupting threshold, to select the most discriminative regions. For these reasons, our proposed method can be regarded as a relatively simple algorithm, which requires only one layer. Unlike most WSOL studies that reported only the performance of the single object localization, we applied our method to a Weakly Supervised Semantic Segmentation (WSSS) task. Since WSSS requires generating pseudo masks for multiple classes and multiple objects, this process allowed us to evaluate how effective our method is for multi-object segmentation. Based on those WSSS results, our proposed method can be used not only for single-object work but also for multi-object work, demonstrating the generalizability of our method.

The main contributions of our work are three-fold:

- We propose a novel Module of Axis-based Nexus Attention (MoANA) that allows us to utilize feature representations from various views in a tensor, thus localizing an object accurately.
- With our proposed calibration of the feature map, our fine-grained attention map adaptively concentrates on the less activated regions along with the class-discriminative regions. Accordingly, it is more likely to focus on informative regions of an entire object in an image.
- Our MoANA achieved the best object localization performances in the metrics of *Top-1 Loc. Err., Top-5 Loc. Err., Gt-known Loc. Err.*, and *MaxBoxAccV2*^22^ on two datasets, i.e., CUB-200-2011^23^ and ILSVRC^24^. Additionally, the segmentation mask generated by employing our MoANA to the WSSS method has the best segmentation performance in the Pascal VOC 2012 dataset^25^.

## Related work

### Weakly supervised object localization

Most of the existing WSOL research addresses corruption methods, which can be categorized into two approaches depending on the strategies of corrupting regions: (1) random corruption^6,11,19^, and (2) network-guided corruption methods^9,10,13,17^.

For the random corruption strategy, Singh and Lee^6^ devised Hide-and-Seek (HaS), an approach that randomly drops patches of input images to encourage the network to find other relevant regions, rather than only focusing on the most discriminative parts of an object. Yun et al.^11^ introduced CutMix, in which the randomly erased (e.g., by cutting) patches are filled with patches of another class, and the corresponding labels are also mixed. Although these methods have been considered as an efficient data augmentation method since they do not require parameters, the random corruption can negatively affect localization performance due to its brute-force elimination of input images.

For the network-guided corruption methods^9,13,17^, the most discriminative regions of the original image or feature map are dropped with a corrupting threshold. Zhang et al.^9^ proposed Adversarial Complementary Learning (ACoL) to find complementary regions through adversarial learning between two parallel-classifiers; one to erase discriminative regions, and the other to learn other discriminative regions except for the erased regions. Choe et al.^13^ introduced an Attention-based Dropout Layer (ADL) that generates a drop mask and an importance map utilizing a self-attention mechanism, and then randomly selects one of them for thresholding feature maps. Mai et al.^17^ proposed Erasing Integrated Learning (EIL) that trains non-discriminative corrupting (e.g., erasing) features and original features with shared CNN layers. However, they all require a corrupting threshold as a parameter for the masking. Our proposed MoANA discovers regions of both class-discriminative regions and non-discriminative but object-related regions using a novel axis-based attention module without the need for a erasing treshold.

There are several other WSOL approaches. SPG^10^ generated a Self-Produced Guidance (SPG) mask for use as pixel-level supervision through attention maps. DANet^12^ employed divergent activation for learning complementary and discriminative visual patterns. NL-CCAM^14^ combined low-probability and high-probability class activation maps. DGL^16^ exploited two kinds of gradients, those of the target class and classification loss. RCAM^15^ alleviated the fundamental problems (e.g., global average pooling, instability of thresholding reference) of the existing CAM^4^ methods by several techniques. $\text {I}^2\text {C}$^18^ leveraged pixel-level similarity with high activation values of two images of the same category. MCIR^19^ utilized two self-attention modules and attention-based fusion loss to get better feature representations. Gao et al.^20^ proposed the Token Semantic Coupled Attention Map (TS-CAM) that employs the self-attention mechanism of visual transformers to mitigate the long-range dependency problem in CNNs and avoid partial activation by generating long-range dependency attention maps. Vitol^21^ employed a patch-based attention dropout layer (p-ADL) in an architecture that utilized a visual transformer for self-attention, expanding the localization map. To the best of our knowledge, most of the above-mentioned WSOL methods have focused on expanding the activated regions, so excessive activated regions were often generated and coarsely localized. Our MoANA can elaborately and naturally expand the activation domain by leveraging various types of discriminative information based on different views of the feature maps.

### Weakly supervised semantic segmentation

Like WSOL, WSSS aims to predict exact pixel-level object masks using weak annotations, a process that requires no expensive labeling. Conventional WSSS methods have trained a classification network with image-level class labels to estimate object localization maps and then employed them as a pseudo mask for semantic segmentation. To do this, most WSSS methods generated the pseudo masks using CAM^4^. However, as CAM is based on intermediate features down-sampled by the classifier, it has issues of poor object localization and incorrect boundary.

To alleviate this problem,^26–28^ focused on expanding incorrect object regions (i.e., seed areas) and^29,30^ attempted to generate better seed areas. Regarding^26–28^, they introduced the seed refinement methods to modify initial seeds obtained from CAM. Kolesnikov et al.^26^ refined CAM by exploiting their Seed, Expand, and Constrain (SEC) principles. Ahn et al.^27^ developed Inter-pixel Relation Network (IRNet) which generates a transition map from the boundary activation map. A Deep Seeded Region Growing (DSRG) network introduced by Huang et al.^28^ found small and subtle discriminative regions from the object of interest using image labels and then produced pixel-level labels.

On the other hand,^29,30^ jointly conducted the pseudo mask generation and segmentation tasks to generate better seeds. Wang et al.^29^ proposed a self-supervised equivariant attention mechanism (SEAM) to narrow the gap between fully and weakly supervised semantic segmentation. Zhang et al.^30^ designed a context adjustment approach (CONTA) which constructs a structural casual model to remove the confounding bias in image-level classification and generate better pseudo-masks as ground truth. We also concentrated on generating better seed areas, however, our MoANA computes fine-level axis-based attentions, and is therefore simple and efficient.

### Attention based deep neural networks

Our MoANA is based on an attention mechanism; therefore, we reviewed existing attention methods even if they were not devised for WSOL. Attention mechanisms have been widely used to enhance the representational power of features. Among various attention mechanisms^31–50^, here, we focused on a context fusion based mechanism^31–33,38,42–46,49,50^ that strengthens the feature maps to be more meaningful by aggregating information from every pixel. For instance, Hu et al.^31^ proposed a Squeeze-and-Excitation Network (SENet) which is a simple and efficient gating mechanism to consider the channel-wise relationships among the feature maps of the basic architectures. Likewise, Woo et al.^32^ devised a Convolutional Block Attention Module (CBAM) that sequentially combines two separate attention maps for channel and spatial dimension. Unlike SENet^31^, CBAM^32^ considered spatial attention which involves “where” to focus. Moreover, to alleviate a limitation of SENet^31^ that utilizes fully-connected layers, Wang et al.^42^ introduced an Efficient Channel Attention Network (ECA-Net)^42^ that deploys a 1D convolutional layer to obtain cross-channel attention, while maintaining lower model complexity.

However, since these methods^31,32,42^ emphasized meaningful features by multiplying the same attention values, where the different information corresponding to spatial (i.e., height and width) or channel dimensions might be ignored, they can be unsuitable for WSOL in which fine location information is demanded. Meanwhile, our MoANA generates a fine-grained attention map that has different attention values across all regions by inferring the connection of channel, height, and width axis-based attention.

## Methods

In this section, we present the details of our proposed Module of Axis-based Nexus Attention (MoANA). MoANA is applied to output feature maps before they are fed into a classifier (Fig. 1) to induce the model to learn the entire region of an object. Hereafter, we regard the output feature maps as a 3D feature tensor, without loss of generality.

**Figure 2:**
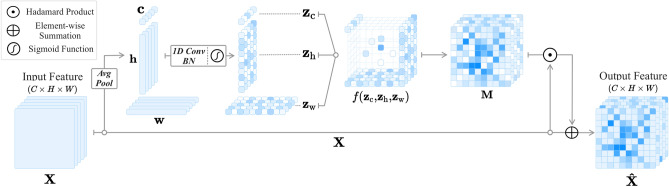
Illustration of Module of Axis-based Nexus Attention (MoANA). An input feature $\textbf{X}$ is processed using triple-view attentions transformed from three kinds of pooled features, $\textbf{c}, \textbf{h}$, and $\textbf{w}$, which are then fed into an expansion function *f*. The generated fine-grained attention map is combined with the input feature, which is referred to as $\textbf{X}\odot \textbf{M}$. A combination of $\textbf{X}\odot \textbf{M}$ and $\textbf{X}$, we obtain $\hat{\textbf{X}}$ is fed it into a classifier.

Our MoANA generates a self-attention tensor derived from three types of view-oriented attention map, by projecting the input feature tensor into the channel, height, and width dimensions, respectively. The MoANA-generated attention tensor presents a fine-grained characteristic in the sense of assigning different attention values for each of the elements in a tensor. The interaction between the complementary information of the axis-based attention matrix in MoANA leads the attention tensor to focus on not only the most discriminative regions, but also on the less discriminative regions of an object. In these regards, the final output feature tensor has an enriched representation resulting in a better object localization output. The overall architecture of the proposed MoANA is illustrated in Fig. 2 and the detailed descriptions are given below.

### Axis-based attention

Let $\textbf{X}\in \mathbb {R}^{C\times H\times W}$ be an input feature tensor, where *C*, *H*, and *W* denote the dimensions of the channel, height, and width, respectively. To condense the global distribution of an input feature tensor $\textbf{X}$ in the triple views, we applied an average pooling in each dimension of the tensor, i.e., channel, height, and width as follows:

1 $$\begin{aligned} \textbf{c} = \text {AvgPool}_\text {w,h}(\textbf{X})\end{aligned}$$

2 $$\begin{aligned} \textbf{h} = \text {AvgPool}_\text {w}(\textbf{X})\end{aligned}$$

3 $$\begin{aligned} \textbf{w} = \text {AvgPool}_\text {h}(\textbf{X}) \end{aligned}$$

where $\text {AvgPool}_{\{\cdot \}}$ is an average pooling operator with respect to the dimensions of $\{\cdot \}$. The three pooled features of $\textbf{c}\in \mathbb {R}^{C\times 1\times 1}$, $\textbf{h}\in \mathbb {R}^{C\times H\times 1}$, and $\textbf{w}\in \mathbb {R}^{C\times 1\times W}$ can be regarded as a summary of the extracted features in $\textbf{X}$ from different viewpoints. Surely, the three views carry different information distributed in the input feature tensor $\textbf{X}$. That is, $\textbf{c}$ captures which feature representations are highly activated, and $\textbf{h}$ and $\textbf{w}$ reflect the discriminative features distributed vertically and horizontally across channels, independently.

Subsequently, in order to utilize their local interaction among units in each pooled feature, we applied a 1D convolution^42^ with a kernel size of *k* and zero-padding without biases, thus keeping their dimensionality. Then, a batch normalization^51^ and a non-linear activation function were applied as follows:

4 $$\begin{aligned} \textbf{z}_\text {c} = \sigma (\text {BN}(\textbf{W}_\text {c}(\textbf{c})))\end{aligned}$$

5 $$\begin{aligned} \textbf{z}_\text {h} = \sigma (\text {BN}(\textbf{W}_\text {h}(\textbf{h})))\end{aligned}$$

6 $$\begin{aligned} \textbf{z}_\text {w} = \sigma (\text {BN}(\textbf{W}_\text {w}(\textbf{w}))) \end{aligned}$$

where $\sigma (\cdot )$ is a sigmoid function and $\textbf{W}_{\{\cdot \}}$ indicates the 1D convolutional layer for the respective pooled features. Here, $\textbf{z}_\text {c}\in \mathbb {R}^{C\times 1\times 1}$, $\textbf{z}_\text {h}\in \mathbb {R}^{C\times H\times 1}$, and $\textbf{z}_\text {w}\in \mathbb {R}^{C\times 1\times W}$ corresponds to the resulting triple-view attentions.

### Attentions expansion

We expanded the triple-view attentions of $\textbf{z}_\text {c}$, $\textbf{z}_\text {h}$, and $\textbf{z}_\text {w}$ to generate an attention map $\textbf{M}\in \mathbb {R}^{C\times H\times W}$ of the same size of the input feature map $\textbf{X}$ by means of an outer sum function *f* as follows:

7 $$\begin{aligned} \textbf{M}&= f(\textbf{z}_\text {c}, \textbf{z}_\text {h}, \textbf{z}_\text {w}) \end{aligned}$$

8 $$\begin{aligned}&= \left[ z_\text {c}^{(i,1,1)}+z_\text {h}^{(i,j,1)}+z_\text {w}^{(i,1,k)}\right] \end{aligned}$$

9 $$\begin{aligned}&= \left[ m^{(i,j,k)}\right] \end{aligned}$$

In Eqs. (8) and (9), $z_\text {c}^{(i,1,1)}, z_\text {h}^{(i,j,1)}, z_\text {w}^{(i,1,k)}$ and $m^{(i,j,k)}$ denotes the elements of each tensor $\textbf{z}_\text {c}, \textbf{z}_\text {h}, \textbf{z}_\text {w}, \textbf{M}$ and *i*, *j*, *k* represent the index of the channel, height, and width dimensions. The values in the attention map $\textbf{M}$ are likely to be different from each other, resulting in a fine-grained attention map. Our fine-grained attention map representation method is different from the previous attention-based methods that learn a coarse attention map having the same values across elements within the same channel. We provide illustrations of tensor-form elements and an example in supplementary B to facilitate a better understanding of our method.

**Figure 3:**
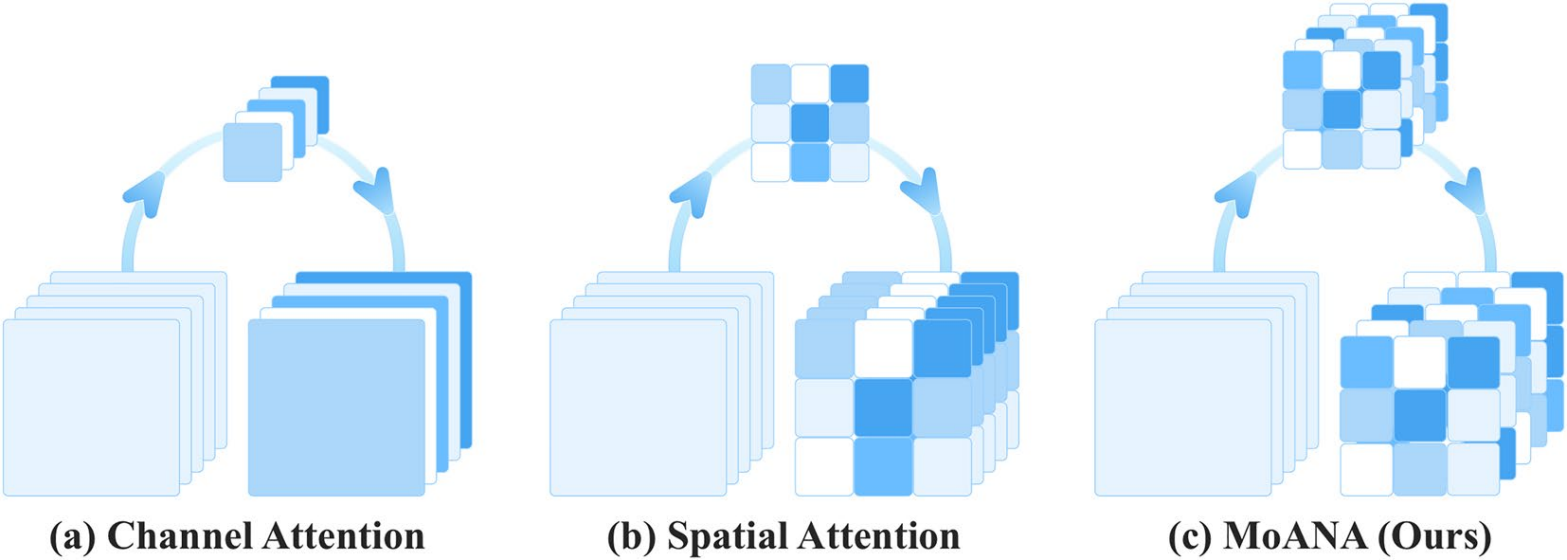
Illustration of conventional context fusion attention approaches and MoANA. Our MoANA calibrates the feature employing an fine-grained attention map generated with axis-based complementary information.

### Feature calibration

We applied the attention tensor estimated in Eq. (7), to the input feature tensor. We considered computational approaches as follows:

10 $$\begin{aligned} \hat{\mathbf {X}}=\textbf{X}\oplus (\textbf{X}\odot \textbf{M}) \end{aligned}$$

where $\odot$ and $\oplus$ denote the Hadamard product and the element-wise summation, respectively.

The proposed approach employs fine-level attention maps, enabling detailed feature calibration at element-level units; this approach is advantageous from the perspective of feature representation learning. The axis-based attended feature ($\textbf{X}\odot \textbf{M}$) that is the sum of discriminative features mined from various viewpoints, has a rich feature representation. Additionally, because the element-wise summation adds an input feature that already contains information about the discriminative feature, it can help to activate regions in which the scaling term is less discriminative.

**Figure 4:**
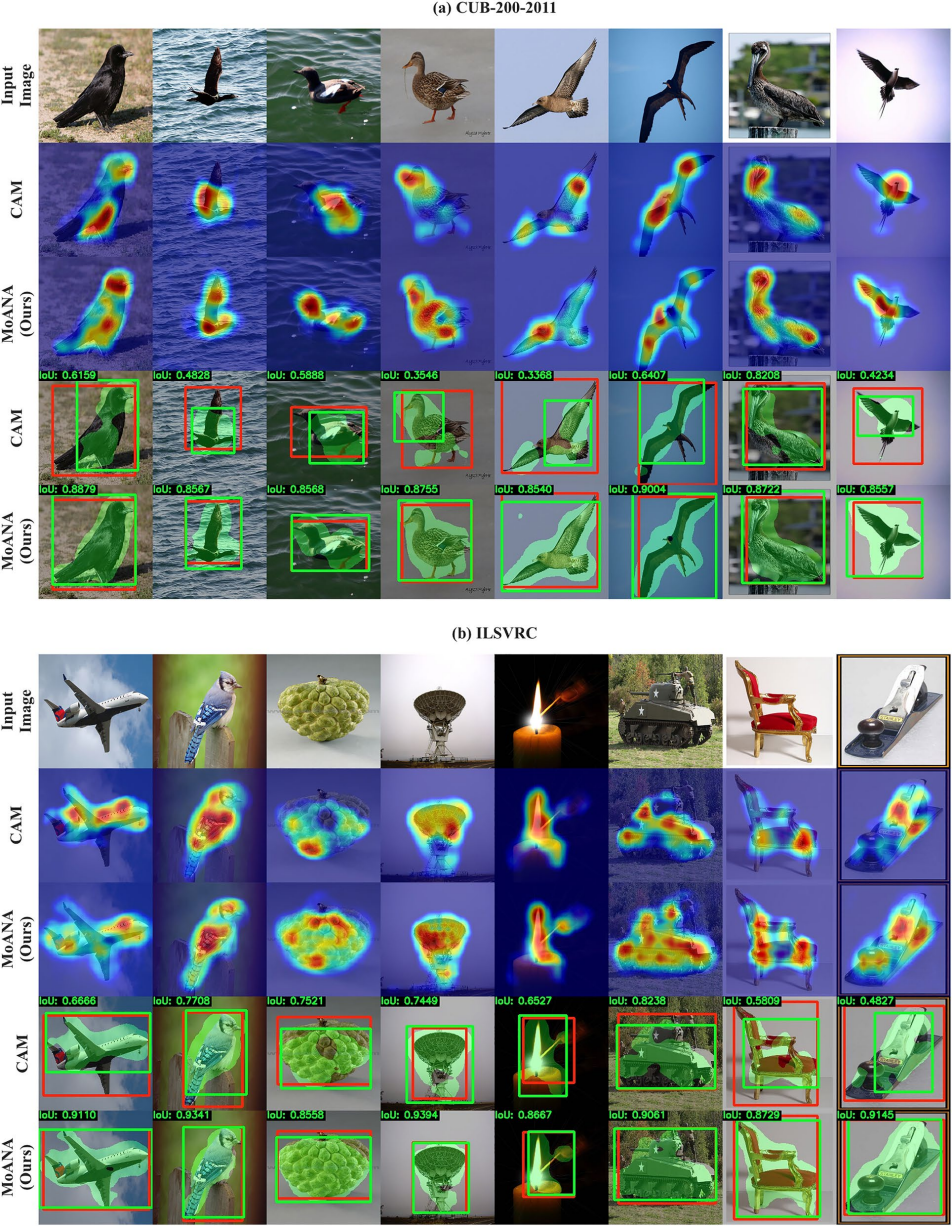
Qualitative comparison between our proposed method (MoANA) and CAM^4^ for WSOL task on the (**a**) CUB-200-2011 and (**b**) ILSVRC datasets. The red box is the GT bounding box, the green box is the predicted bounding box, and the green area is the segmented region to extract the bounding box after the threshold is applied. MoANA can generate more exact localization maps by tightly bounding the entire region of the object in an image. These maps were generated using Python 3.6.0, available at https://www.python.org.

Thus, the attention module described in section “Attentions expansion” is trained to focus not only on the most discriminative features, but also on relatively degraded features. Consequently, our MoANA increases the activation of the object-related regions and relatively lowers the activation of the non-object-related regions. This interpretable phenomenon can be clearly observed from our experimental results in Figs. 4 and 6.

### Distinction to conventional context fusion attention

Figure 3, shows the distinction between the processes of our method and those of other context fusion attention methods. Existing work^31,32,42^ primarily considers channel-wise or spatial-wise attention, ignoring the spatial or channel characteristics distributed over the different maps in a feature tensor. For example, CBAM^32^, one of the representative context fusion attention methods, is used to calculate two attention maps: a spatial attention map with a shape of [$1\times H\times W$], and a channel attention map with a shape of [$C\times 1\times 1$], where *C*, *H*, and *W* are the channel, height, and width. Then, the same attention values are multiplied, ignoring different information between each spatial and channel dimension. Therefore, there still remains a limitation of the context fusion attention mechanism.

Meanwhile, our MoANA method generates three attention maps with [$C\times 1\times 1$], [$C\times H\times 1$], and [$C\times 1\times W$], and then generates a triple-view attention map using the outer sum. The triple-view attention map on different axes provides complementary information not found when only one axis is considered, allowing attention to be paid to fine-grained features not found in existing spatial or channel. Therefore, our MoANA method can calibrate features through the complementary relations inherent in the input feature tensor, thereby achieving the best performance and alleviating a limitation of the context fusion attention mechanism.

## Experiment

### Experiment setup

#### Datasets

We validated our MoANA using three public datasets, CUB-200-2011^23^ and ILSVRC^24^ for WSOL and Pascal VOC 2012^25^ for WSSS. CUB-200-2011 includes a total of 11, 788 images from 200 bird categories, divided into 5, 994 images for training and 5794 images for evaluation. ILSVRC consists of 1.2 million images in about 1000 categories for training and 50, 000 images for a validation. Pascal VOC 2012 contains a total of 21 classes, composed of 1, 464 training images, 1449 validation images and 1456 test images. In our experiments, we used the 10, 582 training images generated by^52^, and 1449 validation images.

#### Competing methods

We compared our MoANA with the existing state-of-the-art WSOL methods, CAM^4^, HaS^6^, ACoL^9^, SPG^10^, CutMix^11^, ADL^13^, NL-CCAM^14^, RCAM^15^, DGL^16^, EIL^17^, and $\text {I}^2\text {C}$^18^. In order to observe the effectiveness of our methods in WSSS, we compared it with five other WSSS methods, SEC^26^, DSRG^28^, IRNet^27^, CONTA^30^, and SEAM^29^

.

#### Evaluation metric

For quantitative evaluation, we used the *Top-1 Loc. Err.*, *Top-5 Loc. Err.*, and *Gt-known Loc. Err.* metrics. *Top-N Loc. Err.* is the fraction of images that the IoU between the predicted bounding box and the ground truth bounding box is less than 50%, and the target class does not exist in the *N* classes with the highest class prediction probability. *Gt-known Loc. Err.* is the fraction of images that the IoU between the predicted bounding box and the ground truth (GT) bounding box is less than 50%, regardless of the classification result. We additionally used the recently proposed metric *MaxBoxAccV2*^22^ over the IoU thresholds $\delta \in \{0.3,0.5,0.7\}$ at the optimal activation map threshold. A threshold of the activation map, $\tau$, was set between 0 and 1 at 0.01 intervals. Our final results of *MaxBoxAccV2* measured various localization performances over threshold $\tau$ for activation maps at various levels of $\delta$. In semantic segmentation, quantitative evaluation was performed using the mIoU score.

**Table 1:** Quantitative results compared to other WSOL methods using Top-1, Top-5, Gt-known localization errors on the CUB-200-2011 and ILSVRC datasets. A lower value is an indicator of better performance.The best performance is highlighted in bold, and the second-best performance is underlined.

Methods	Backbone	CUB-200-2011			ILSVRC		
		Top-1 $\downarrow$	Top-5 $\downarrow$	Gt-Known $\downarrow$	Top-1 $\downarrow$	Top-5 $\downarrow$	Gt-Known $\downarrow$
CAM^4^	VGG16	55.9	47.8	44.0	57.2	45.1	38.9
HaS^6^	InceptionV3	58.9	–	42.3	50.3	–	34.5
ACoL^9^	VGG16	54.1	43.5	40.7	54.2	40.6	37.0
SPG^10^	GoogLeNet	53.4	42.3	37.3	51.4	40.0	35.3
CutMix^11^	VGG16	47.5	–	28.2	56.6	–	36.1
ADL^13^	InceptionV3	47.0	–	36.7	51.3	–	38.4
DANet^12^	GoogLeNet	47.5	38.0	–	52.5	41.7	–
NL-CCAM^14^	VGG16	47.6	35.0	–	49.8	39.3	34.8
RCAM^15^	VGG16	41.0	–	23.7	55.4	–	39.3
DGL^16^	VGG16	43.9	–	–	52.3	–	35.2
EIL^17^	VGG16	42.5	–	26.2	53.2	–	29.7
$\text {I}^2\text {C}$^18^	InceptionV3	44.0	31.7	27.4	46.9	35.9	31.5
MCIR^19^	VGG16	41.9	–	–	48.4	–	33.7
CAM^4^	ResNet-50	50.6	46.4	26.8	53.7	40.0	37.2
ACoL^9^	ResNet-50	42.2	–	27.3	52.6	–	38.4
SPG^10^	ResNet-50	48.5	–	28.4	51.5	–	36.6
CutMix^11^	ResNet-50	45.2	–	32.2	52.8	–	34.6
ADL^13^	ResNet-50	37.7	–	26.5	51.5	–	35.9
RCAM^15^	ResNet-50	40.5	–	22.4	50.6	–	37.8
DGL^16^	ResNet-50	39.2	29.5	–	46.6	37.3	33.5
$\text {I}^2\text {C}$^18^	ResNet-50	37.6	–	17.4	45.2	35.4	31.5
MCIR^19^	ResNet-50	35.3	–	22.7	47.6	–	32.1
TS-CAM^20^	DeiT-S	**28.7**	**16.2**	22.3	46.6	35.7	32.4
Vitol^21^	DeiT-S	–	–	–	46.3	–	**28.2**
MoANA (Ours)	ResNet-50	32.9	19.6	**15.8**	**45.2**	**34.9**	31.9

**Table 2:** Quantitative results comparing other WSOL methods with *MaxBoxAccV2*^22^ using Resnet-50 as backbone. A higher value is an indicator of better performance.

Methods	MaxBoxAccV2 $\uparrow$	
	CUB-200-2011	ILSVRC
CAM^4^	63.0	63.6
HaS^6^	64.7	63.4
ACoL^9^	66.5	62.2
SPG^10^	60.4	63.2
CutMix^11^	62.8	63.2
ADL^13^	58.4	63.6
MoANA (Ours)	**71.4**	**65.8**

### Implementation details

#### Weakly supervised object localization

We used a ResNet-50^53^ pre-trained with ILSVRC as the backbone network. In order to obtain localization maps, we used $1\times 1$ convolutional layers, similar to ACoL^9^. For the kernel size *k* in the axis-based attentions, we used 3, according to^42^. The input images of training were resized to $256\times 256$ and then we cropped $224\times 224$ patches randomly from the resized images. Then, they were flipped horizontally with a probability of 0.5. The test images were resized to $224\times 224$. For the ILSVRC dataset, we trained our MoANA using a stochastic gradient descent (SGD) optimizer with a momentum of 0.9, weight decay of 0.0005, and a mini-batch size of 256 for 20 epochs. The learning rate was decreased from initial values of 0.002 for the feature extractor and 0.02 for the remaining modules by multiplying by 0.1 after at every 5 epochs. For the CUB-200-2011 dataset, we set a mini-batch size of 32 for 45 epochs, an initial learning rate of 0.01, and a learning rate decay rule of multiplying by 0.1 every 10 epochs.

#### Weakly supervised semantic segmentation

We used IRNet^27^ as the base model to generate a Pseudo-Mask. We trained by feeding MoANA to the classification network of IRNet. We used the pseudo-masks generated using IRNet, as GT, to train the segmentation network DeepLab v2^54^ for WSSS. The input image was transformed through the same process as IRNet: horizontal flipping, random cropping, and color jittering. The classification model was trained with the input image cropped as $512\times 512$ and 16-sized batches. We used a weight decay with a coefficient of 0.0001, an SGD optimizer with a momentum of 0.9, and an activation map threshold of 0.16. A total of 8000 iterations were trained, starting with an initial learning rate of 0.1 and using polynomial decay, which is $lr_{init} = lr_{init}(1-itr/\max _{itr})^{0.9}$ at every iteration. All settings were the same as in DeepLab v2^54^, except that the segmentation model setting used a pseudo-mask as the GT label. We implemented all methods in PyTorch and trained with Titan X GPU. The Code is available at: https://github.com/ku-milab/MoANA.

### Experimental results

#### Weakly supervised object localization

We visualize the predicted localization bounding boxes and activation maps for the CAM^4^ and MoANA methods in Fig. 4. We also indicate the IoU value between the predicted bounding box and the GT box at the upper left corner. We observed that MoANA elaborately localized the entire part of an object for CUB-200-2011 and ILSVRC datasets. While CAM^4^ focused on the partial objects or covered the outside of the exact object region, MoANA tightly bounded the entire region of the object in an image, thereby achieving the best localization performance.

Table 1 summarize the localization performance of the competing methods. In Table 1, we observed the effectiveness and reliability of our MoANA in localization tasks, consistently achieving the best or second-best performance in various evaluation localization metrics on the CUB-200-2011 and ILSVRC. In Table 2, MoANA achieved the best *MaxBoxAccV2*^22^, of 71.4 for CUB-200-2011 and 65.8 for ILSVRC, evaluated at the optimal activation map threshold.

**Figure 5:**
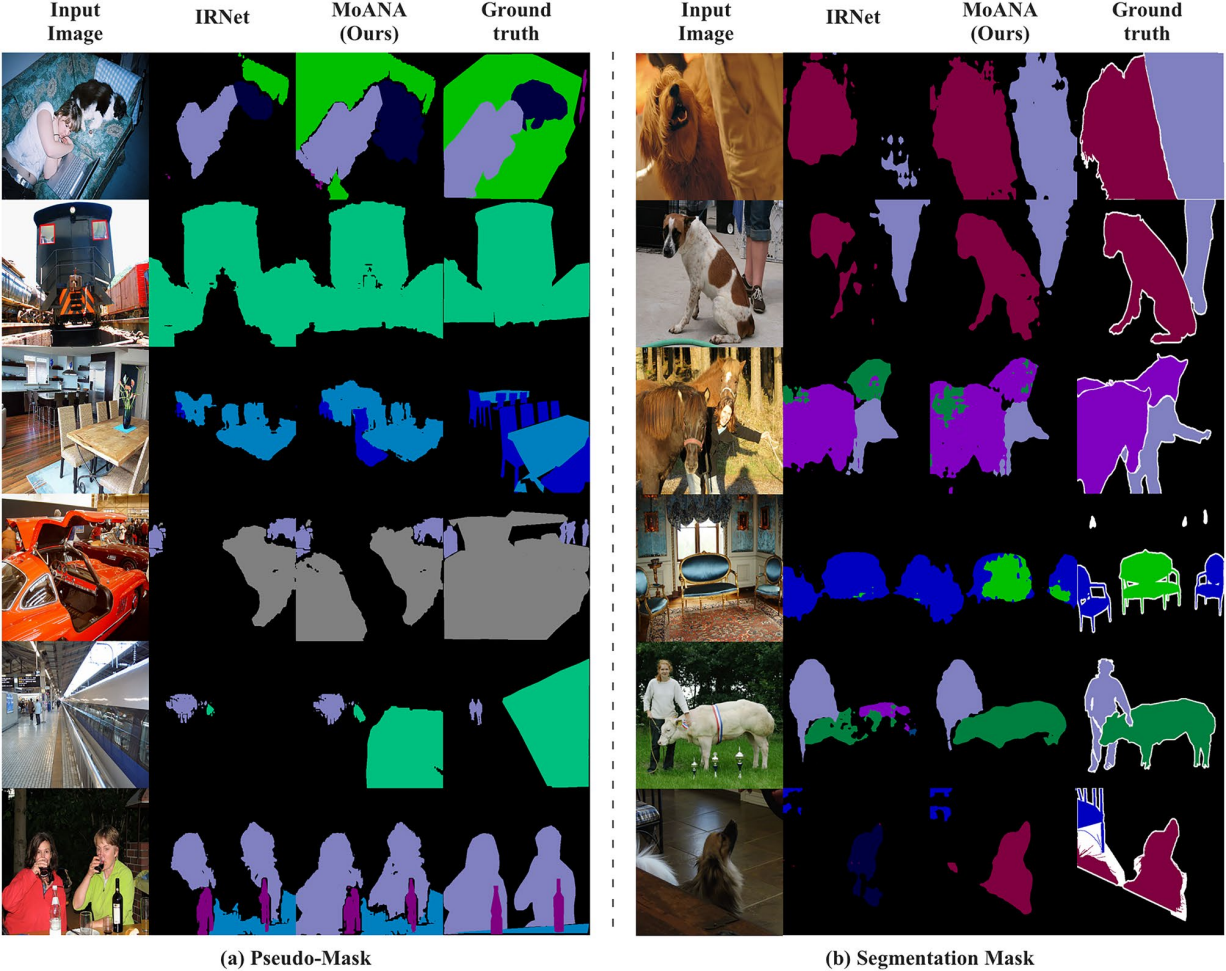
Qualitative comparison between our proposed method (MoANA) and IRNet^27^ for WSSS task on the Pascal VOC 2012 dataset. Multi-label regions were segmented to be more similar to GT than IRNet^27^.

**Table 3:** Quantitative results to other WSSS methods using mIoU on the Pascal VOC 2012 dataset.

Methods	Backbone	mIoU (%)		
	(for Pseudo-mask)	CAM	Pseudo-mask	Seg. mask
Fully supervised				
DeepLab v2^54^	–	–	–	**77.7**
Weakly supervised				
SEC^26^	VGG16	46.5	53.4	50.7
SEAM^29^	ResNet-38	**55.1**	63.1	64.3
DSRG^28^	ResNet-101	47.3	62.7	61.4
IRNet^27^	ResNet-50	48.3	65.9	63.0
SC-CAM^55^	ResNet-101	50.9	–	66.1
BES^56^	ResNet-101	50.4	67.2	65.7
CONTA^30^	ResNet-50	48.8	67.9	65.3
CDA^57^	ResNet-50	50.8	67.7	65.8
AdvCAM^58^	ResNet-101	55.6	69.9	68.1
RIB^59^	ResNet-101	56.5	70.6	68.3
PPC^60^	ResNet-38	**61.5**	70.1	67.7
RECAM^61^	ResNet-101	54.8	**70.9**	68.5
SIPE^62^	ResNet-101	58.6	69.2	**68.8**
MoANA (Ours)	ResNet-50	49.2	66.7	67.0

The best performance is highlighted in bold.

**Table 4:** WSOL Results of MoANA about combination approach on the CUB-200-2011 and ILSVRC datasets.

Methods	Datasets	Loc.Err (%)		
		Top-1	Top-5	Gt-Known
(1) $$\hat{\mathbf {X}}=\textbf{X}$$				
CAM^4^	CUB	55.9	47.8	44.0
	ILSVRC	57.2	45.1	38.9
(2) $$\hat{\mathbf {X}}=(\textbf{X}\odot \textbf{M})$$				
MoANA w/o combination	CUB	34.0	23.0	18.8
	ILSVRC	45.8	35.1	**31.9**
(3) $$\hat{\mathbf {X}}=\textbf{X}\oplus (\textbf{X}\odot \textbf{M})$$				
MoANA (Ours)	CUB	**32.9**	**19.6**	**15.8**
	ILSVRC	**45.2**	**34.9**	**31.9**

The best performance is highlighted in bold.

#### Weakly supervised semantic segmentation

We visualize the semantic segmentation results for Pascal VOC 2012^25^ are shown in Fig. 5. Specifically, Fig. 5a illustrates the pseudo-mask generated by the classification model, and Fig. 5b shows the segmentation mask obtained from the segmentation model trained with the pseudo-mask as the segmentation label.

In Fig. 5a, an analysis of the outcomes produced by IRNet illustrates that the pseudo-masks are confined to distinct sections of each object, a challenge reminiscent of the issues inherent in CAM in WSOL. However, a distinct transformation is observed when our proposed method is applied; the mask’s scope extends, covering the entirety of the objects. A case in point can be observed in the 6th row of Fig. 5a, where the traditional approach is centered on prominent features, such as the facial region of a person. In contrast, our technique expands the mask to cover the entire bodily structure.

In Fig. 5b, the influence of our enhanced pseudo-masks on the accuracy of segmentation masks is demonstrated. The segmentation model learned with the IRNet-based pseudo mask shown in Fig. 5a can identify the problem of segmenting only certain parts of the object or segmenting into the wrong class. The segmentation model learned with the pseudo mask generated by applying our method expands the object area and is accurately classified. This is particularly evident in the 5th row of Fig. 5b, where specific sections of the cow are initially misclassified, and the correctly identified areas are confined. However, the integration of our method not only corrects the misclassifications but also augments the segmented mask area to align more precisely with the ground truth. In other words, compared to baseline methods, MoANA effectively identifies and corrects missed segment regions, resulting in representations that are more closely aligned with the actual ground truth.

Table 3 summarizes the results of MoANA and the competing methods in the fully and weakly supervised settings for Pascal VOC 2012. When we employed the MoANA method as a module into IRNet, although the performance did not exceed that of the most advanced methods, a notable enhancement in mIoU was observed. These results underscore the potential applicability of our method in contexts involving multi-label and multi-object tasks. Detailed mIoU results for each class are show in supplementary A.

### Analysis and ablation study

#### Effect of feature combination approach

In order to investigate the combination feature map effect, we compared the results with and without the combination approach in Eq. (10) in terms of the localization and segmentation task. Based on an understanding of a combination operation, note that $\textbf{X}\oplus (\textbf{X}\odot \textbf{M})$ leads the function $\textbf{X}\odot \textbf{M}$ to learn information that the input feature tensor $\textbf{X}$ may have missed or emphasized less. The ablation study was conducted by dividing the investigation into three cases: (1) original features, (2) calibration features by scaling with attention value, and (3) calibration features as a combination of scaling features and input features (Table 4). We demonstrated the effectiveness of the combination approach by observing that our proposed method performed best in the three cases.

**Figure 6:**
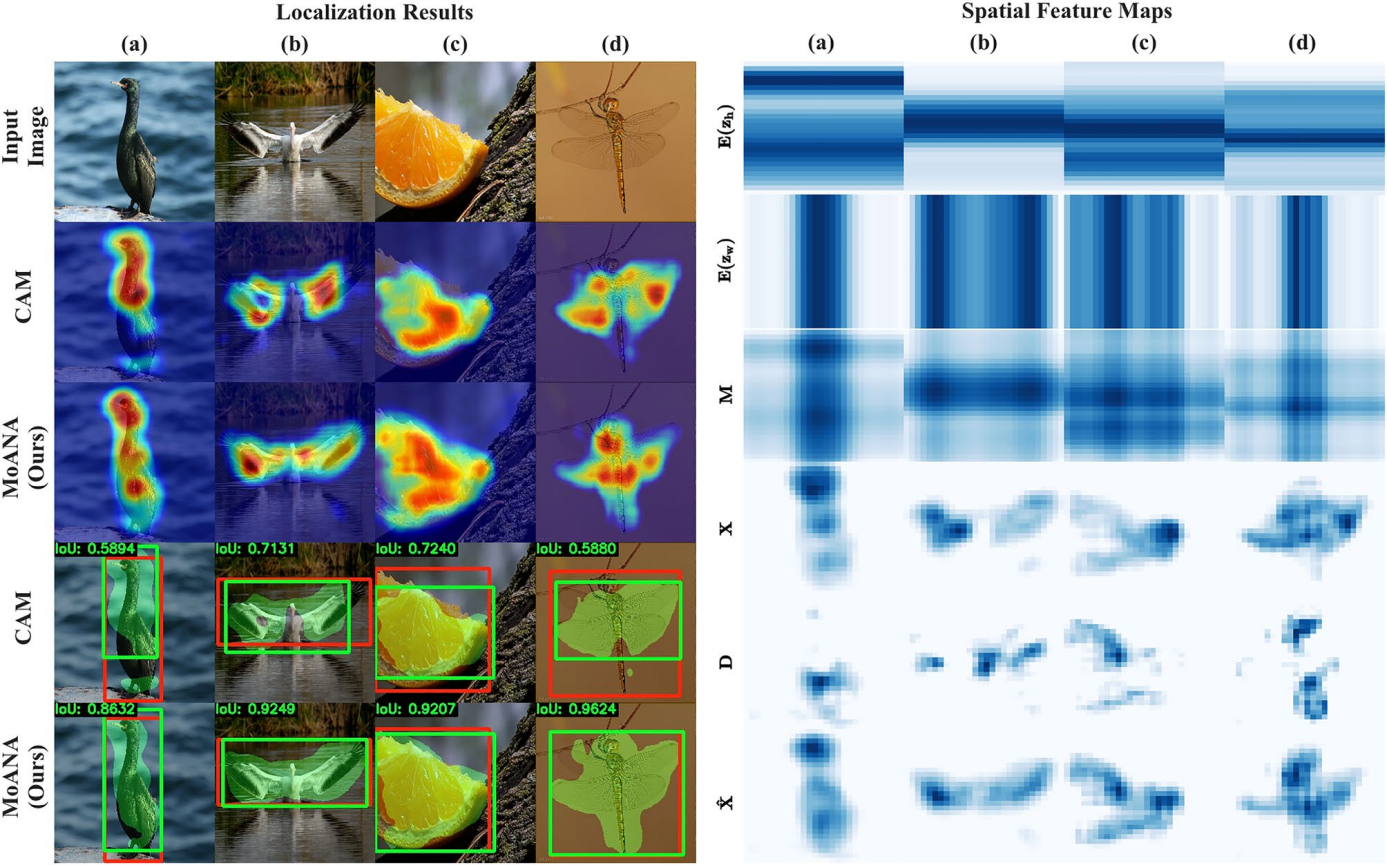
(Left) Visualization of activation maps and bounding boxes in CAM^4^ and our MoANA for comparison. (Right) We plotted triple-view attention maps ($\mathbf {E(z_h), E(z_w)}$ and $\textbf{M}$) in our MoANA by normalizing them in a range between 0 and 1. Here, $\mathbf {E_{\{\cdot \}}}$ indicates the expansion of the pooled feature to the input feature size. Also, we plotted the normalized difference $\textbf{D}$ between $\textbf{X}$ and $\hat{\textbf{X}}$ to show to which MoANA gives attention. If the column names are the same in the left and right figures, the input image is the same. These maps were generated using Python 3.6.0, available at https://www.python.org.

#### Visualization of attention map

To get an insight into the working of our MoANA, we visualized the axis-based attention maps $\mathbf {{z}_{h}}$ and $\mathbf {{z}_{w}}$, the combined attention map $\textbf{M}$, the input feature map $\textbf{X}$, the resulting output feature map $\hat{\textbf{X}}$, and the difference $\textbf{D}$ between $\textbf{X}$ and $\hat{\textbf{X}}$ in Fig. 6. We transformed the expanded attention map $\mathbf {E(z_h)}$ and $\mathbf {E(z_w)}$ into a matrix by channel-wise average pooling, for visualization. However, the attention map of $\textbf{z}_c$ is omitted because there was no difference in the values of the map when channel-wise average pooling was performed. We normalized each matrix in the range of [0, 1].

From the Fig. 6 of localization results, we observed an activation map where the CAM focuses only on the part of the object regions, such as wings. On the other hand, MoANA generates sophisticated activation maps by paying additional attention to activated object regions such as wings and inactivated object-related regions such as bodies. Furthermore, it can be observed that the body and wing regions are calibrated regions in the $\textbf{D}$ row of the spatial feature maps column (b) of Fig. 6. From the viewpoint of attention map generation, the role of $\textbf{X}\odot \textbf{M}$ can be interpreted as being to excite the less activated regions in which the target task-related information is inherent. As shown in Figs. 4 and 6, we validated the effectiveness of our fine-grained calibration of features in WSOL.

## Conclusion

In this paper, we proposed a novel Module of Axis-based Nexus Attention (MoANA) to accurately localize an object in an image. MoANA consists of three components; (i) triple-view attentions, (ii) an expansion of the attentions, and (iii) calibration of the features. Our proposed method utilizes complementary information from axis-based attention for the calibration of sophisticated object-related regions within the feature map. MoANA therefore does not require an additional hyperparameter such as a corrupting threshold for masking the discriminant regions in the corrupting methods. Our proposed method achieved the highest performance in localization and segmentation tasks in terms of *Top-1 Loc. Err., Top-5 Loc. Err., Gt-known Loc. Err., Seg. Mask mIoU,* and *MacBoxAccv2* metrics over three datasets. Our experimental results show the validity of all three components and interpreted the inner working of the feature calibration. Our proposed method can be plugged into any CNN architecture without modifying the original network architecture, in the sense that we applied MoANA on the final output of the feature extractor before a classifier. Further, we applied our algorithm to the WSSS task of multi-object localization. In that sense, it would be our forthcoming research issue to more generalize its application to various CNN tasks (e.g., object detection).

### Supplementary Information

Supplementary Information.

## Data Availability

We have evaluated our proposed method on the CUB-200-2011, ILSVRC, and Pascal VOC 2012 dataset. All datasets are publicly available, and more information can be found at the following link: (CUB-200-2011) https://www.vision.caltech.edu/datasets/cub_200_2011/, (ILSVRC) https://www.image-net.org/challenges/LSVRC/, (Pascal VOC 2012) http://host.robots.ox.ac.uk/pascal/VOC/voc2012/.

## Code availability

All codes used in our experiments are available at https://github.com/ku-milab/MoANA.
