# Supplementary material for: Module of Axis-based Nexus Attention for weakly supervised object localization
Source: Sci Rep. 2023 Oct 30;13:18588. doi: 10.1038/s41598-023-45796-8 (PMC10616293; doi:10.1038/s41598-023-45796-8)
Supplement: Supplementary file 1 — Supplementary Information. [file 41598_2023_45796_MOESM1_ESM.pdf]

Module of Axis-based Nexus Attention for Weakly Supervised Object Localization

Junghyo Sohn[1], Eunjin Jeon[2], Wonsik Jung[2], Eunsong Kang[2] and Heung-Il Suk[1,2,*]
[1]Department of Artificial Intelligence, Korea University, Seoul 02841, Republic of Korea
[2]Department of Brain and Cognitive Engineering, Korea University, Seoul 02841, Republic of Korea
*Corresponding author: hisuk@korea.ac.kr

Supplementary A.

Table S.1 shows the mIoU of the segmentation mask per class. There were 17 classes superior to baselines out of a total of 21 classes. We demonstrated generalization by observing the better performance of the proposed method in most classes.

Table S.1. Segmentation performance (mIoU) for each class on Pascal VOC 2012 dataset.

**Class**	**Methods**	
	IRNet	**MoANA (Ours)**
Background	89.2	**89.7**
Aeroplane	**72.2**	68.2
Bicycle	29.4	**30.7**
Bird	**80.7**	78.9
Boat	59.7	**61.3**
Bottle	**66.5**	65.7
Bus	87.9	**88.1**
Car	79.5	**79.7**
Cat	76.8	**81.5**
Chair	29.5	**30.0**
Cow	75.9	**79.9**
Dining table	38.0	**40.5**
Dog	70.1	**77.0**
Horse	76.0	**77.0**
Motorbike	75.6	**77.1**
Person	72.9	**74.1**
Potted plant	49.3	**51.2**
Sheep	78.9	**80.1**
Sofa	41.9	**45.0**
Train	70.6	**72.5**
TV/monitor	**60.7**	58.4

Supplementary B.

Fig. S.1 and Fig S.2 illustrate the attention values of each element in tensor to aid in understanding Eq. (8). Each tensor corresponds to $\mathbf{z}_\mathrm{c} \in \mathbb{R}^{C\times 1\times 1}, \mathbf{z}_\mathrm{h} \in \mathbb{R}^{C\times H\times 1}, \mathbf{z}_\mathrm{w} \in \mathbb{R}^{C\times 1\times W}$, and $\mathbf{M} \in \mathbb{R}^{C\times H\times W}$. The i, j, k represent the indices of arbitrary channel, height, and width dimensions, respectively.

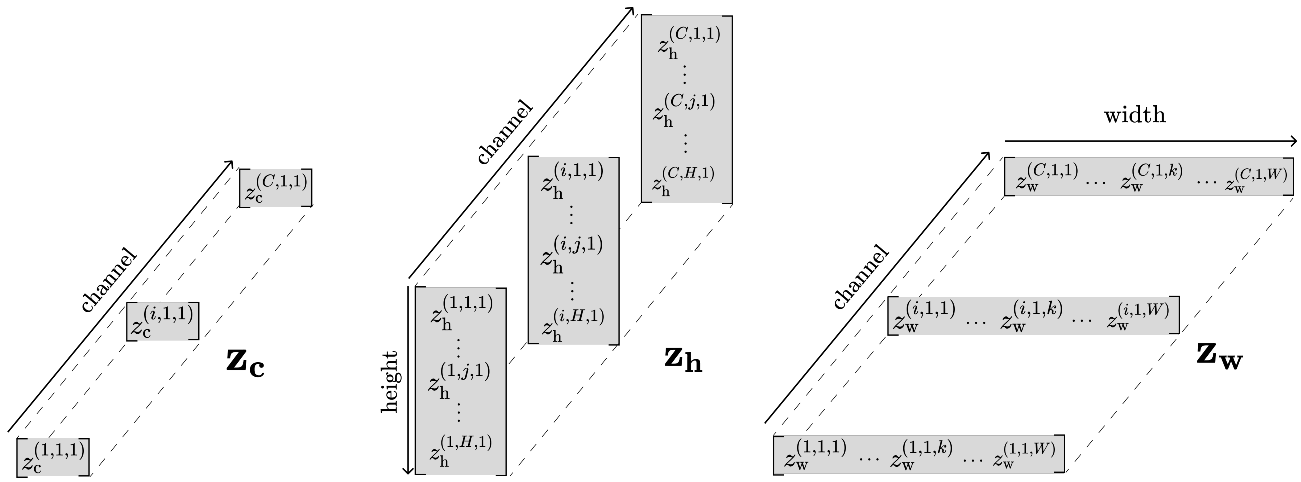

Figure S.1. Illustrating the elements of the attention map for the channel, height, and width axis in tensor.

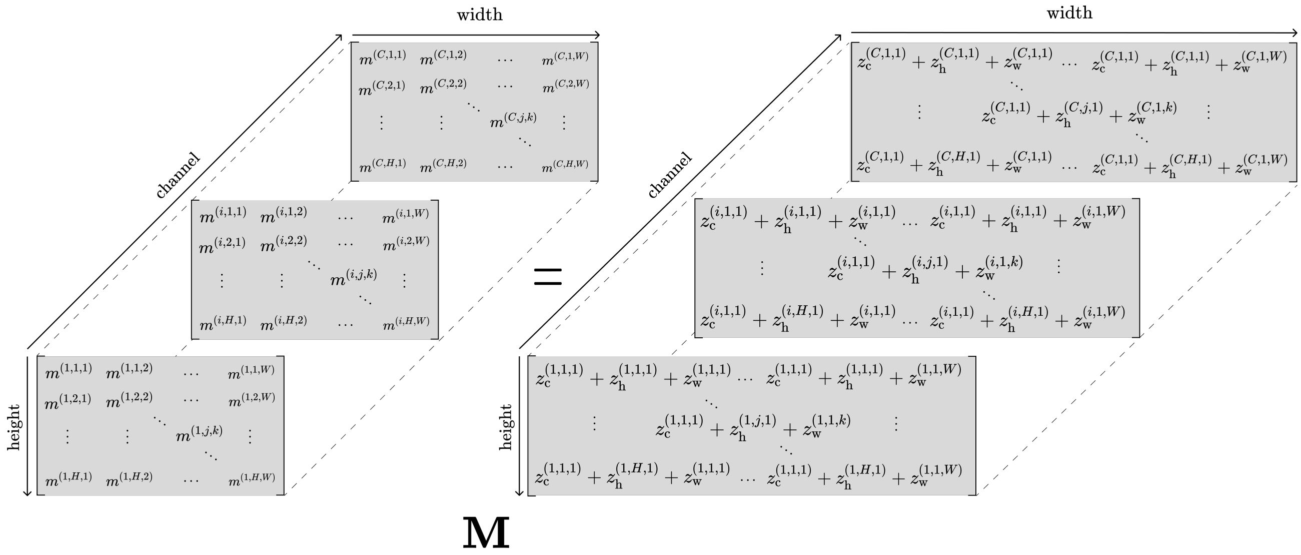

Figure S.2. Illustrating the elements of the final attention map in tensor.

To facilitate a clearer understanding, we provide an example where $C = H = W = 2$. This example illustrates the complex interactions within our model, offering insights into the contributions of each component to the overall result. Each tensor corresponds to $\mathbf{z}_\mathrm{c} \in \mathbb{R}^{2\times 1\times 1}, \mathbf{z}_\mathrm{h} \in \mathbb{R}^{2\times 2\times 1}, \mathbf{z}_\mathrm{w} \in \mathbb{R}^{2\times 1\times 2}$, and $\mathbf{M} \in \mathbb{R}^{2\times 2\times 2}$.

Example)

$$\mathbf{z}_\mathrm{c} = \left(\begin{pmatrix} 2 \\ 3 \end{pmatrix} \right) \mathbf{z}_\mathrm{h} = \left(\begin{pmatrix} 5 \\ 4 \end{pmatrix} \begin{pmatrix} 9 \\ 6 \end{pmatrix} \right) \mathbf{z}_\mathrm{w} = \left(\begin{pmatrix} 3 & 8 \\ 1 & 5 \end{pmatrix} \right) \tag{1}$$

$$M = \left(\begin{array}{c} \begin{pmatrix} z_{\mathrm{c}}^{(1,1,1)} + z_{\mathrm{h}}^{(1,1,1)} + z_{\mathrm{w}}^{(1,1,1)} & z_{\mathrm{c}}^{(1,1,1)} + z_{\mathrm{h}}^{(1,1,1)} + z_{\mathrm{w}}^{(1,1,2)} \\ z_{\mathrm{c}}^{(1,1,1)} + z_{\mathrm{h}}^{(1,2,1)} + z_{\mathrm{w}}^{(1,1,1)} & z_{\mathrm{c}}^{(1,1,1)} + z_{\mathrm{h}}^{(1,2,1)} + z_{\mathrm{w}}^{(1,1,2)} \end{pmatrix} \\ \begin{pmatrix} z_{\mathrm{c}}^{(2,1,1)} + z_{\mathrm{h}}^{(2,1,1)} + z_{\mathrm{w}}^{(2,1,1)} & z_{\mathrm{c}}^{(2,1,1)} + z_{\mathrm{h}}^{(2,1,1)} + z_{\mathrm{w}}^{(2,1,2)} \\ z_{\mathrm{c}}^{(2,1,1)} + z_{\mathrm{h}}^{(2,2,1)} + z_{\mathrm{w}}^{(2,1,1)} & z_{\mathrm{c}}^{(2,1,1)} + z_{\mathrm{h}}^{(2,2,1)} + z_{\mathrm{w}}^{(2,1,2)} \end{pmatrix} \end{array} \right) \tag{2}$$

$$= \left(\begin{array}{c} \begin{pmatrix} 2+5+3 & 2+5+8 \\ 2+4+3 & 2+4+8 \end{pmatrix} \\ \begin{pmatrix} 3+9+1 & 3+9+5 \\ 3+6+1 & 3+6+5 \end{pmatrix} \end{array} \right) = \left(\begin{array}{c} \begin{pmatrix} 10 & 15 \\ 9 & 14 \end{pmatrix} \\ \begin{pmatrix} 13 & 17 \\ 10 & 14 \end{pmatrix} \end{array} \right) \tag{3}$$

$$= \left(\begin{array}{c} \begin{pmatrix} m^{(1,1,1)} & m^{(1,1,2)} \\ m^{(1,2,1)} & m^{(1,2,2)} \end{pmatrix} \\ \begin{pmatrix} m^{(2,1,1)} & m^{(2,1,2)} \\ m^{(2,2,1)} & m^{(2,2,2)} \end{pmatrix} \end{array} \right) \tag{4}$$

The example shows that when aggregating attention values across each axis to compute the final **M**, distinct attention values are assigned to each pixel, enabling a more fine-grained representation.

Supplementary C.

Fig. S.3 illustrates three attention maps generated by (a) the channel attention method[1], (b) the spatial attention method[2], and (c) our proposed method for the input image, respectively. To provide a more detailed view of each channel's matrix, we have visually represented the attention across axes $\mathbf{E}(z_c), \mathbf{E}(z_h), \mathbf{E}(z_w)$, as well as the final attention map $\mathbf{M}$ in Fig. S.4.

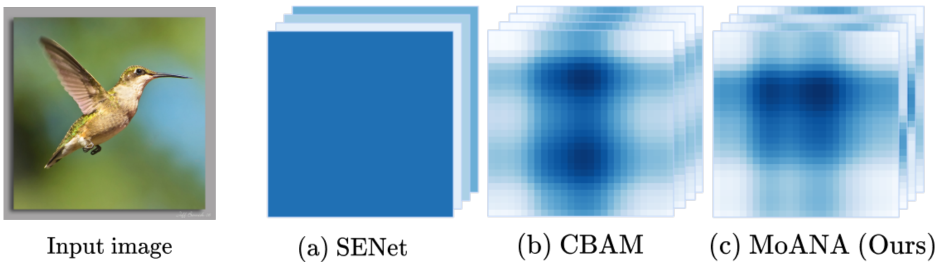

Figure S.3. Illustration of visualizing (a) channel attention map, (b) spatial attention map, (c) fine-grained attention map (Ours) for the feature map. These maps were generated using Python 3.6.0, available at `https://www.python.org`.

Observing the coarse-grained and fine-grained attention tensors in Fig. S.3-(a), the same attention value is applied to all pixels within a single channel, and in Fig. S.3-(b), the same attention value is applied across all channels for a single pixel. In contrast, our proposed fine-grained attention applies distinct attention values to each channel and pixel.

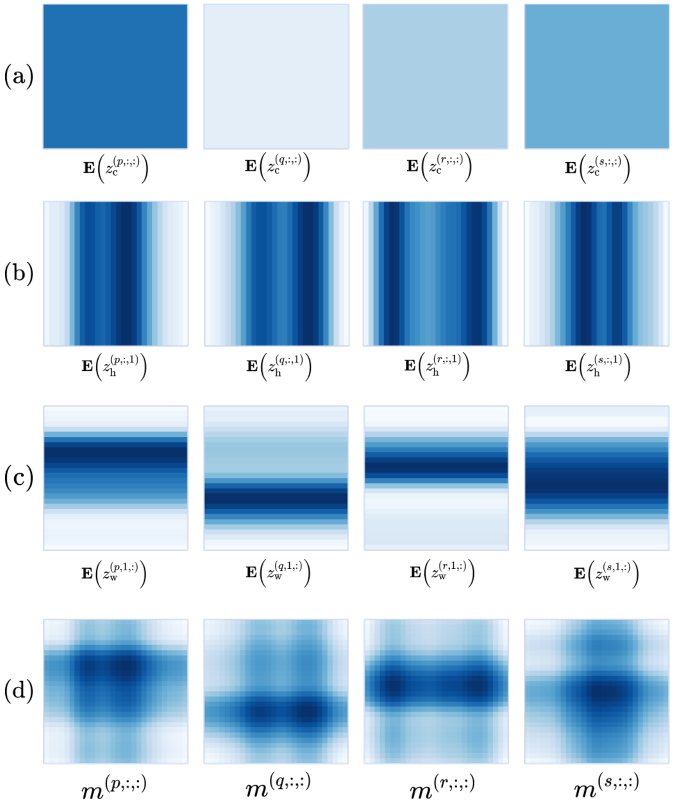

Figure S.4. Visualize the attention values of each element in each arbitrary channel (*i.e.*, p, q, r, s) from Fig. S.3-(c). $\mathbf{E}(z)$ means extending the attention values of each axis into a matrix form. These maps were generated using Python 3.6.0, available at `https://www.python.org`.

In Fig. S.4, we detail the construction of $m^{(i,:,:)}$, where $i \in \{p, q, r, s\}$, corresponding to each channel illustrated in Fig. S.4-(d). The attention maps for each channel, height, and width are depicted in Fig. S.4-(a), Fig. S.4-(b), and Fig. S.4-(c), respectively, each presented in matrix form. This depiction unequivocally demonstrates that $m^{(i,:,:)}$ is derived from these respective attention maps.

References

1. Hu, J., Shen, L. & Sun, G. Squeeze-and-excitation networks. In *Proceedings of the IEEE Conference on Computer Vision and Pattern Recognition*, 7132–7141 (2018).

2. Woo, S., Park, J., Lee, J.-Y. & Kweon, I. S. CBAM: Convolutional block attention module. In *Proceedings of the European Conference on Computer Vision*, 3–19 (2018).
